# Barriers and facilitators of HIV treatment services among men who have sex with men during COVID-19 lockdown

**DOI:** 10.4102/sajhivmed.v26i1.1670

**Published:** 2025-04-24

**Authors:** Betty Sebati, Edith Phalane, Amukelani Bilankulu, Refilwe N. Phaswana-Mafuya

**Affiliations:** 1 South African Medical Research Council/University of Johannesburg Pan African Centre for Epidemics, Research Extramural Unit (SAMRC/UJ PACER EMU), Faculty of Health Sciences, University of Johannesburg, Johannesburg, South Africa; 2 Anova Health Institute, Polokwane, South Africa

**Keywords:** HIV treatment services, programme implementers, men who have sex with men, COVID-19 lockdown, Consolidated Framework for Implementation Research

## Abstract

**Background:**

The provision of HIV treatment services was severely impacted by the COVID-19 pandemic and the subsequent lockdown measures, particularly among men who have sex with men (MSM), a population disproportionately affected by HIV.

**Objectives:**

To explore the service providers’ perspectives on the barriers and facilitators of the HIV treatment services during the COVID-19 lockdown in Capricorn District, Limpopo province.

**Method:**

The study followed an exploratory design and was conducted in Capricorn District in Limpopo province. A purposive sample of 10 HIV treatment service providers were included in the study. An interview guide was developed using the Consolidated Framework for Implementation Research (CFIR) domains and associated constructs. The transcribed qualitative data were captured and analysed on Atlas.ti version 24.

**Results:**

The barriers included fear of COVID-19 transmission, movement restrictions during the initial phase of the lockdown period, target-driven performance pressure, lack of mobile clinics, and understaffing. The facilitators included teamwork among the various stakeholders in the programme, tailoring strategies to reach more MSM, partnerships and connections with the Department of Health and other relevant organisations.

**Conclusion:**

The study revealed that the tailoring of the MSM programme facilitated access to HIV treatment services during COVID-19.

**What this study adds:** This study adds insights into the barriers and facilitators of providing HIV treatment services among MSM. It emphasises the importance of programme adaptability and partnerships for a multisectoral effort for sustainable HIV services under conditions similar to the COVID-19 lockdown.

## Introduction

South Africa has the highest HIV prevalence in the world, with approximately eight million people living with HIV (PLWH).^1^ The country has made significant progress in combatting HIV in the last few years. Some of this progress includes the adoption of the Joint United Nations Programme on HIV/AIDS (UNAIDS) 95-95-95 targets (i.e., 95% of PLWH diagnosed, 95% of those diagnosed to be on antiretroviral therapy [ART], and 95% of those on ART to be virally suppressed) by the year 2025.^2,3,4^ These goals were set for the advancement of HIV prevention and treatment services.^3,5^ As of 2022, South Africa’s progress towards the 95-95-95 goals was that 90% of people were diagnosed with HIV, 91% of those diagnosed were on ART, and 94% of those on ART were virally suppressed among adults aged 15 years and older.^3^

COVID-19 lockdown measures posed challenges to the provision of and accessibility to healthcare services. In South Africa, lockdown measures were put in place after the declaration of the national state of disaster on 15 March 2020. These included restrictions on travelling and social distancing, which led to the closure of most public places such as schools, restaurants, and other businesses regarded as non-essential.^6,7^ Fear of COVID-19 transmission also negatively impacted on routine HIV clinic access, and a sharp downturn in HIV testing during the lockdown likely reduced the number of potential ART initiation candidates. The literature points to different experiences in HIV service outcomes during the lockdown period. Pillay et al.^8^ found that the COVID-19 lockdown led to a decrease in HIV testing. However, Siedner et al.^9^ reported an overall improvement in HIV services outcomes, including HIV testing and ART adherence. A decrease in the utilisation of HIV services during the COVID-19 lockdown was reported among key populations: men who have sex with men (MSM), female sex workers, incarcerated individuals, and people who inject drugs.^10,11^ This is of great concern, as key populations are disproportionately affected by the HIV epidemic.^12^ They are at an increased risk of HIV acquisition and transmission; they also experience homophobia, stigma, discrimination and violence, further limiting their access to HIV services.^13,14^

There is a limited number of qualitative studies assessing the impact of COVID-19 on HIV services among MSM in South Africa; qualitative studies reviewed were conducted elsewhere.^15,16^ Quantitative studies done in South Africa and elsewhere showed a significant decrease in HIV-related services; in particular, HIV testing and ART initiations were heavily impacted by the COVID-19 lockdown, while ART provision was largely maintained.^10,17,18,19^

The current study focuses on service providers’ perspectives on the barriers and facilitators of the HIV treatment services during the COVID-19 lockdown in Capricorn District, Limpopo province. Service providers played a pivotal role as frontline workers to ensure the continuity of HIV services during the pandemic period. The study strengthens qualitative knowledge on the subject matter towards improved understanding of the administration of HIV treatment services during a crisis like the COVID-19 pandemic.

## Research methods and design

### Study design

The current study followed an exploratory design,^20^ to qualitatively explore service providers’ perspectives on the barriers and facilitators of HIV treatment services during the COVID-19 lockdown in Capricorn District, Limpopo province. This was accomplished through semi-structured service provider interviews guided by the Consolidated Framework for Implementation Research (CFIR), which is an analytical framework with proven effectiveness in providing comprehensive context-specific determinants of health service innovations, including views and perspectives. In local settings, it is a commonly utilised framework for evaluating potential facilitators and barriers.^21,22^ The facilitators and barriers to HIV services were identified using CFIR constructs. This enabled a theory-informed analysis.^21^ The framework is made up of five domains (innovation, inner setting, outer setting, individual, and implementation process domains), each divided into constructs.^22^

### Study site

This study was conducted in Capricorn District Municipality based in Limpopo province, in the north-eastern part of South Africa. It is made up of four local municipalities (Blouberg, Molemole, Polokwane, and Lepelle-Nkumpi).^23^ Being the third-largest district in the province, it makes up 12% (185 222.27 hectares) of Limpopo province’s total surface area, comprising 28 traditional authorities, 113 wards, and a total population of 1 372 355. It accounts for approximately 23% of the province’s population, and 2.3% of the country’s population.^23^ This is where the MSM programme was being implemented.

### Description of the men who have sex with men programme

The MSM programme was implemented by Beyond Zero, from 2019 to date, across nine districts of six provinces in South Africa (Capricorn and Mopani districts in Limpopo province; Oliver Tambo District in the Eastern Cape; Mangaung District in the Free State; King Cetshwayo, Ugu, and Uthukela districts in KwaZulu-Natal; Gert Sibande District in Mpumalanga; and Bojanala Platinum District in North-West). The aim of the programme was to prevent and reduce infections and transmissions of HIV, tuberculosis (TB) and sexually transmitted infections (STI) among MSM, with an estimated sample size of 52 635 MSM across all districts and provinces. The MSM programme is funded by the Global Fund. It provides the following services to MSM: HIV testing, HIV prevention services (e.g. condoms, pre-exposure prophylaxis [PrEP], post-exposure prophylaxis [PEP]), lubricants, STI and TB screening and treatment, and HIV treatment services including linkage to HIV care, ART initiation, ART adherence, retention in care, and viral load testing. The MSM programme mainly delivered these services through outreach activities in MSM hotspots, by invitation, or through walk-ins into their facilities. This study only focused on the MSM programme implemented in Capricorn District, Limpopo province, which served over 6000 MSM during the COVID-19 pandemic period. Service provision was disrupted by the COVID-19 lockdown for the first three months of lockdown (March–May 2020), during which time the MSM service providers could not go to work or physically reach MSM prior to receipt of travel permission letters from the Department of Health. This current study focused on the HIV treatment services offered through the above-mentioned MSM programme in Limpopo province.

### Study population and sample size

The study followed a comprehensive approach involving interviewing implementation team members with various roles and responsibilities to get an extensive view of their experiences, barriers, facilitators, and opportunities for improvement of the MSM programme. We purposively selected 10 service providers who were strongly engaged in the implementation of the MSM programme during COVID-19. These included professional and enrolled nurses, HIV testing services (HTS) counsellor and retention officer, social workers, liaison officers, outreach workers and team leaders.

The MSM community contact person, who is also part of the non-profit organisation offering the MSM programme, assisted with the recruitment by inviting and introducing the researcher to the service providers. Interested participants were invited to take part in the study. Only service providers who were engaged in the implementation of the MSM programme during the COVID-19 lockdown and provided written consent to participate in the study were included in the analysis.

### Data collection

Guided by the CFIR domains and associated constructs,^22^ an interview guide was developed (Online Appendix 1). The interview guide included information about how COVID-19 impacted the day-to-day running of the MSM programme, whether alterations have been made to ensure effective implementation, whether they were aware of the needs and preferences of MSM during the COVID-19 lockdown period and what was done to meet them. It further included questions on the identified barriers faced by MSM in participating in the MSM programme, the programme goals and how progress towards them was assessed among other factors. Probing questions were utilised where necessary to get a more thorough understanding of the responses. The interviewees reflected on the COVID-19 lockdown period from 26 March 2020, when alert level five began in South Africa, to 31 December 2021, when it was downgraded to alert level one. All interviews were conducted in English between 26 February and 15 March 2024, and lasted 25–60 min. All the interviews were conducted on a one-on-one basis in a private room where the MSM programme activities take place. All the interviews were audio-recorded and transcribed verbatim before analysis. Table 1 shows the domains and constructs that the current study focused on.

**TABLE 1:** The Consolidated Framework for Implementation Research domains and associated constructs included in the current study.

Domains	Constructs
Innovation	Source and evidence base
	Relative advantage
	Complexity
	Cost
Outer setting	Critical incidents
	Partnerships and connections
	Policies and laws
	Performance measurement pressure
Inner setting	Work infrastructure
	Communications
	Available resources
Implementation process	Teaming
	Planning
	Tailoring strategies

*Source*: Adapted from Damschroder LJ, Reardon CM, Widerquist MA, Lowery J. The updated Consolidated Framework for Implementation Research based on user feedback. Implement Sci. 2022;17(1):75. https://doi.org/10.1186/s13012-022-01245-0

### Data analysis

The transcribed qualitative data were captured on Atlas.ti version 24.^24^ Codes were derived independently by two coders upon a comprehensive overview of the data for familiarisation. This was guided by both inductive coding and the CFIR domains and constructs. The two coders later discussed and finalised the codes. Thematic content analysis was utilised to derive patterns from the created codes, guided by the objective of the study.

### Ethical considerations

This study has been reviewed and approved by the Research Ethics Committee (REC) of the University of Johannesburg (UJ), South Africa (REC-1949-2023), and the MSM community gatekeeper in Capricorn District, Limpopo province. All participants provided written consent for taking part in the interviews. Qualitative data containing the personal information of the participants were de-identified. The interview recordings were saved in a password-protected online platform with controlled access, while hard copy consent forms are locked inside a cabinet in an access-controlled office at the UJ.

## Results

Table 2 shows the characteristics of the study participants. The current study included 10 service providers who assumed various roles in the implementation of the MSM programme during the COVID-19 lockdown. Their ages ranged from 26 to 47 years old, with a median age of 36.6 years. Only three of the participants were women, while seven were men. Two of the participants were outreach team leaders, two were outreach workers, one was the programme liaison officer, one was an HTS and retention counsellor, one was a social worker, and three were nurses (one enrolled and two professional nurses). The participants have been in the programme for various years ranging from 1.5 to 10 years.

**TABLE 2:** Characteristics of the service providers in the programme.

Service providers	Sex	Age (years)	Role in the programme	Years working in the programme
Service provider 1	Male	38	Outreach team leader	4.5
Service provider 2	Male	37	Outreach team leader and driver	10.0
Service provider 3	Male	37	Liaison officer	6.0
Service provider 4	Male	26	Outreach worker	5.0
Service provider 5	Female	37	Social worker	3.5
Service provider 6	Male	47	Enrolled nurse	4.5
Service provider 7	Male	35	Outreach worker	4.5
Service provider 8	Female	40	Professional nurse	4.0
Service provider 9	Female	28	Professional nurse	1.5
Service provider 10	Male	41	HTS and retention counsellor	4.0

HTS, HIV testing services.

### HIV treatment services among men who have sex with men during the COVID-19 lockdown period

When the lockdown restrictions started, they led to a halt in all programme activities, while service targets remained unchanged at the onset of the pandemic:

‘Yoooh, COVID-19, the worst thing that you can talk about. The COVID-19 disturbed us in terms of the service, the activities, everything was stopped at that time. The activity was stopped, uhm on the other side the target remains like that. Then we came up with a strategy.’ (Service provider 1, 38 years old, male)

### Facilitators of access to HIV treatment services during the COVID-19 lockdown period

#### Funding availability

The Global Fund was cited as the source and funder of the MSM programme. The MSM programme was implemented in the Capricorn District because of factors such as the high migration to the area, and the high concentration of tertiary institutions and job opportunities. Furthermore, the district includes the capital city of the Limpopo province, which is Polokwane city. Additionally, the city has a higher prevalence of HIV in the district and high HIV risk among MSM:

‘I believe it’s been implemented because of the previous research that has been conducted showing that men who are having sex with other men are at high risk of HIV. So that’s why the programme is implemented specifically in this district. There is a high prevalence of HIV in the district, the reason being you get a lot of your tertiary institutions, the migration of students, they’re in and out of the district. People come, people go. So each and every year there are people who are coming. We don’t know what they are coming with. If you can check the whole of Limpopo, this is the city. Polokwane is the city. So everyone from the whole of Limpopo, we are looking for greener pastures here in Polokwane.’ (Service provider 3, 37 years old, male)

#### Focus on men who have sex with men HIV care services

During the COVID-19 lockdown, the MSM programme played an essential role in providing focused HIV services, effectively attending to the unique needs of MSM that were overlooked given that public health facilities were primarily concerned with the public at large. The MSM programme emerged as the district’s preferred provider of HIV care for MSM, providing tailored support during a period of increased difficulties:

‘I am not bad-mouthing the Department of Health, but it was easy if we go collect the MSM and they get assisted by us. Their hands were tied, it was COVID so they could not just assist anyone … I won’t say it was a new thing but there was no organisation that was providing these services. So, people will always compliment us because it’s something that they never expected one day there will be a specific programme for the MSM. At this stage, we are still leading. We don’t know as time goes on because things change.’ (Service provider 2, 37 years old, male)

#### Partnerships with various stakeholders

The MSM programme had formed partnerships and connections with various stakeholders to support the implementation of the programme. The major partnership was with the Department of Health, wherein newly diagnosed MSM found through the programme were linked with the Department of Health facilities for ART initiation, ART collection, and adherence counselling. Moreover, this partnership had become very useful for the MSM programme during the COVID-19 lockdown through the provision of a permission letter that allowed the programme leaders to travel and reach MSM ensuring continuity of services. Additionally, buddies within communities assisted with reaching new MSM during the lockdown when outreach was not possible:

‘The Department of Health and “us” made an agreement that for those people who are due for treatment and have an emergency, they will give us letters so that we can connect and take them to the facility. We also have these people from the communities that we call “buddies”. We want someone who is famous and knows many MSMs, so they are one of the people that assist us in recruiting new clients.’ (Service provider 2, 37 years old, male)

#### Constant communication on men who have sex with men-friendly and other social media platforms

In addition to communicating with the MSM through phone calls, SMSs, face-to-face, and social media platforms such as WhatsApp and Facebook, the MSM programme also communicated with the MSM through MSM-friendly online dating platforms such as Badoo and Grindr. These platforms became useful during the COVID-19 lockdown, particularly for those MSM who did not have personal contact details of the programme staff and could not physically come to the programme offices to access services because of the pandemic. The staff utilised these to share updates on the programme activities with the MSM across the district:

‘We also used social media platforms. We have a gay-friendly platform, Grindr. You must also check it, Grindr. The Grindr [*sic*] is only for MSM. So, when you go there, we don’t ask you if you are MSM. We see it, we communicate with the MSM, and the people that are next to them. So, when we are there, we update our Grindr, then we start to sell out [*sic*] services. There is another one they call Badoo, we had a person, a linkage officer with a work phone, who posted there that today we will be at this place then if you want PrEP we are there, if you want to test, we are there.’ (Service provider 1, 38 years old, male)

#### Resource availability

During the COVID-19 lockdown, the MSM programme had some resources which allowed them to mitigate the impacts of the lockdown. For instance, the availability of cars allowed service providers to travel to various sub-districts in one day to offer the MSM services such as transport to a public facility for ART collection. Even though it was costly on the programme compared to their expenditure in normal circumstances, it was beneficial to the MSM:

‘You know, when you have materials there is no place which is far. So that was an advantage to our side. We were driving from Polokwane to Zebediela, Polokwane to Mafefe, Polokwane to Lebowakgomo, Polokwane to Ga Dikgale, that way we were doing it. On the other side, it was good for the client, but on the other side it was not good because we were consuming a lot of petrol, even the budget was going high.’ (Service provider 1, 38 years old, male)

#### Multidisciplinary team; teamwork

The team members involved in the implementation and delivery of the MSM programme exhibited a variety of skills and professions for a comprehensive service provision ranging from nurses (both enrolled and professional), retention and HTS counsellors, linkage officers, social workers, outreach personnel and leaders, among others. Although each of the team members had their roles and responsibilities, during the COVID-19 lockdown they all worked together as a team and certain responsibilities were for all; these included recruiting new clients, and encouraging the MSM to stay on the programme, or assisting with any challenges they may have been facing:

‘If they say we must reach 30, we must reach it regardless of whether there is COVID or not. So, it’s when as a team we came up with a plan on what best we can do. Yes, we could not all move. It was not all of us, it was the team leaders only. We were three by that time. So, it was us using one car. So, it means that if I am tired then the other drives, we go to another site. Each of us was having their own list. It means that we had three lists. We were ensuring that all the clients in the lists are reached because we were supposed to report at the end of the day the activities that we were doing so that it would support the petrol consumption.’ (Service provider 1, 38 years old, male)

‘Basically, we do have ambassadors, we’ve got HTS counsellors, retention counsellors and linkage officers, but all of us are doing the same job. We don’t say this is your job. It’s your job when it gets to the end, but in terms of recruiting, in terms of giving courage, we all participate in the same way. As I can say is that this office has to do the same job but at the end of the day, everyone takes his duties to the end.’ (Service provider 10, 41 years old, male)

#### Guidance from managers, funders and other stakeholders through performance reviews

The MSM programme-planning activities were mainly motivated by the performance reviews, wherein they met monthly and quarterly to review their progress towards the targets given by the funder, then plan a way forward to meet and exceed their targets and further brainstorm on how to improve their services to reach more MSM, and for the MSM to have the best experience in the programme despite the challenges brought by COVID-19:

‘Every month we have meetings. We display the targets and then display the number obtained. If we feel like we haven’t reached the target this month, we make sure that we give each other ideas of how to reach it in the following month.’ (Service provider 4, 26 years old, male)

‘We were having quarterly reviews where we go out of the office, and they booked somewhere where we review. It’s us in Capricorn and Mpumalanga, and we all gather and discuss our performance, discuss our challenges, our plans going forward and all that.’ (Service provider 9, 28 years old, female)

#### Implementation of innovative strategies

The daily activities involved in the implementation and delivery of the MSM programme had to be altered because of the COVID-19 pandemic and the resultant lockdown. The MSM programme team had operationalised strategies to address the barriers encountered in the COVID-19 lockdown context. One of the strategies implemented was for the programme to use their cars to transport MSM due for ART from their homes to the facility, and back home. Another strategy was referred to as a ‘safe space’, wherein MSM who needed HIV testing and had a safe and confidential space to conduct the test would reach out to the programme to come and test them in a space that felt safe to them. Despite all the above-implemented strategies, some of the MSM did not come for their follow-up appointments and defaulted on ART, probably because of their fear of COVID-19:

‘During the COVID-19 lockdown, we used cars. We made sure that we called and checked that this person was due for the treatment, and then that’s when we used our travel permission letter to collect the beneficiary and take them to the facility. So, it means that if it was not us, they would default.’ (Service provider 1, 38 years old, male)

‘Maybe after six or seven months of the COVID-19 lockdown that’s when we started to implement “safe space”. We go to a certain area, there is someone there. If that person has a safe space, then we test them because at that time we were not allowed to put our gazebos in public since everything was on hold. So at least with safe space, it was working.’ (Service provider 2, 37 years old, male)

### Barriers to HIV treatment services during the COVID-19 lockdown period

#### COVID-19 movement restrictions; lack of a mobile clinic; antiretroviral medication theft

Although some resources were availed in an effort to ensure the MSM programme continuation, the implementation of the programme faced several challenges during the COVID-19 lockdown, including movement restrictions and lack of mobile clinics to enable timely provision of HIV treatment. The availability of mobile clinics would have enabled the programme to safely store (i.e., within recommended temperatures) and distribute ART during outreach without having to transport MSM to the health facility. This would have reduced the time taken for MSM to get ART and reduced petrol expenditure for the programme. However, safety issues for ART theft were also a concern, leading to further complications:

‘We cannot carry ART with us in the outreach because of the temperature outside. And also, in the country that we are in, crime is high. And we are thinking about the drugs. Yeah. So if they know that you are carrying an ART drug with you, they can come and hijack you.’ (Service provider 1, 38 years old, male)

#### Relocation of men who have sex with men to remote areas leading to increased transport costs

Although the services offered by the MSM programme were free of charge, the COVID-19 pandemic had resulted in the relocation of some programme beneficiaries to remote areas. This led to increased costs of the programme, because of having to travel to the MSM to provide the required services:

‘You find that a client is supposed to go collect his medication at the facility but with the COVID-19 regulations that [*sic*] nobody was allowed to move around. That’s where it was costing us, because if you find that a client stays within the district but far from Polokwane, we would go to that client, fetch them with our company car, and take them to the facility to get his treatment. So, my point is that it was costly even in terms of time. And you would find that our clients are staying far apart from each other, that’s how it was costing us. It was very hectic at that time.’ (Service provider 7, 35 years old, male)

#### The men who have sex with men programme was not regarded as an essential service during the lockdown

During the COVID-19 lockdown, policies surrounding movement restrictions caused the greatest impact on the delivery of the MSM programme. These included remaining indoors except when seeking essential services such as food and urgent medical assistance. MSM were not able to access and utilise the services provided while initially, the programme staff were also unable to travel to MSM and had to temporarily close their offices:

‘Our office was closed during the COVID-19 lockdown. Our clients used to come here for follow-up. So that time we were unable to see them.’ (Service provider 6, 47 years old, male)

#### Target-driven performance; failure to reach targets due to the lockdown

Although the main goal and driver of the MSM programme was to provide HIV services and education to MSM and assist the Department of Health in ending HIV as a pandemic, it was also target-driven. The MSM programme was funding-dependent hence it performed under pressure to reach the given targets to retain the funding. If targets were not met within the given period, a backup plan was needed to ensure targets were met. The COVID-19 lockdown has temporarily led to disruptions in meeting some of the targets. One of the targets during the COVID-19 lockdown was to reach 500 MSM for HIV testing. There were no targets for ART; it was dependent on the number of MSM tested for HIV:

‘We have our database that we use to divide the targets. We check how many MSM we have in the database, if we see that the database does not have the number of the target it means that we see how we can come up with a plan to reach additional numbers. Then we organise workshops, Pride events etc. It was difficult to do so during the lockdown since gatherings were not allowed. If we don’t reach the target, the money will go.’ (Service provider 1, 38 years old, male)

#### Understaffing

Although the MSM programme team was doing their best in their capacity to ensure continuity of services for MSM during the COVID-19 lockdown, they were limited by being understaffed, with nearly 20 team members serving almost 6000 MSMs:

‘We are limited. You see the team, we are less than 20 but how many clients do we have, it’s more than 6000, so we are limited.’ (Service provider 3, 37 years old, male)

**FIGURE 1:**
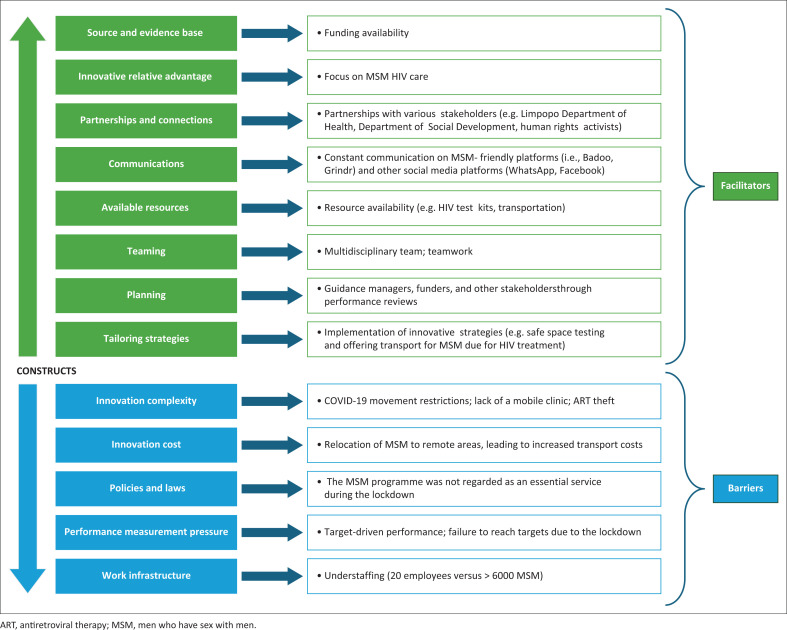
Barriers and facilitators of the men who have sex with men programme during the COVID-19 lockdown.

Figure 1 shows the barriers and facilitators identified in the study and the associated CFIR constructs.

## Discussion

The study explored the service providers’ perspectives on the barriers and facilitators of HIV treatment services during the COVID-19 lockdown in Capricorn District, Limpopo province. Barriers identified were fear of COVID-19 transmission, movement restrictions, target-driven performance pressure, lack of mobile clinics, and understaffing. In line with the CFIR model, the facilitators included teaming, motivation, and commitment of team members, tailoring strategies to reach more MSM, and partnerships and connections with the Department of Health and other relevant organisations. Similarly, Nitpolprasert et al.^15^ identified accessible ART refills, online support interventions, and local HIV service delivery responses as facilitators for accessing HIV services and for optimum ART adherence.

The COVID-19 pandemic has demonstrated the importance of teamwork as a facilitator for the provision of HIV services, wherein team members had to work together to meet the targets. Despite all the challenges experienced, including fear of contracting COVID-19, the MSM programme service providers continued to stay motivated and committed to serving MSM during the COVID-19 lockdown. Partnerships and connections with external parties such as the Department of Health, Department of Social Development, human rights organisations, and other key population organisations facilitated the successful implementation of the MSM programme during the COVID-19 lockdown. This corroborates with a South African study by Naidoo et al.,^25^ which reported that key multisectoral stakeholders are pivotal for the adoption of programme implementation. Another study reported improved collaboration as one of the significant changes resulting from the COVID-19 pandemic.^26^

The COVID-19 lockdown measures served as barriers to the provision of HIV services by the programme at the beginning of the pandemic, impacting the complexity, cost, and performance of the programme. In terms of the CFIR model, this relates to the innovation and outer setting domains. These barriers provided an opportunity for the MSM programme to implement innovative strategies to improve its services and reach more MSM. In line with the findings by Hergaty et al.,^27^ the programme-implementing team in the current study had to tailor their daily activities and implement strategies to facilitate the continuity of the MSM programme. Jaafari et al.^28^ found that PLWH in Iran experienced financial difficulties and alterations to their care plan. Tailored strategies were implemented, including ‘safe space’ wherein MSM had a safe personal space for HIV testing, and the transportation of MSM due for ART refills to the healthcare facility. The MSM programme also relied on ‘buddies’. Buddies are a representative group of MSM from various sub-districts within the Capricorn District who facilitated continued service provision by informing the programme service providers on the needs and preferences of the MSM during the COVID-19 lockdown. A study by Wulandari et al.^29^ conducted among HIV health workers in Indonesia reported the implementation of a multi-month dispensing strategy for ART, and home-based ART delivery services during the same period. This is supported by Wilkinson and Grimsrud,^30^ who depicted that the COVID-19 pandemic and its associated barriers had presented an opportunity for innovative implementations to scale up HIV services. The barriers to ART services during the COVID-19 pandemic may have led to the emergence of opportunistic infections, drug resistance, co-morbidities, and increased death.^31,32,33,34^

The findings identified through this CFIR approach may be utilised to improve the implementation of the MSM programme in similar conditions.

### Strengths and limitations

The possibility of recall bias cannot be excluded from the study, since the interviews were conducted approximately 4 years after the lockdown in South Africa. The results may not be generalisable to other settings, particularly to HIV treatment services in public health facilities and HIV services in other countries. Despite the above, this study has contributed valuable insights into the impact of COVID-19 on HIV treatment services among MSM from the service providers’ perspectives. Consolidating the reported information with insights from the MSM and other service providers can be very fundamental in the improvement of HIV treatment services. Moreover, it has contributed to the knowledge of the barriers and facilitators shared by non-government HIV programme implementors for possible intervention by the government, policymakers and other relevant stakeholders.

## Conclusion

The study revealed that tailoring of the MSM programme facilitated access to HIV treatment services during COVID-19. Access was provided through the establishment of safe spaces for HIV testing, transporting MSM to health facilities, teaming, partnerships and connections (e.g. with the Department of Health). Lessons learnt can be used to empower HIV service providers on how to sustain HIV treatment or similar services during pandemic times.
